# Foreign Summary

**Published:** 1840-04-25

**Authors:** 


### FOREIGN SUMMARY.

### PHILIPS' LECTURE ON THE PRINCIPLES AND PRACTICE OF SURGERY.—NO. II.

Scrofula—(Continued.)

*Most probable Causes considered—Treatment—Change of Climate—Food—Exercise—Medical Means—Purgatives—Emetics—Blood-letting—Alkalies—Muriate of Baryta, Iodine internally, externally—Mercurials—Cicatrices.*

CANCER.

*Nature—Anatomical Characters—Varieties—After Carswell, after Müller—Scirrhoid Structures—Anatomically—Chemically—Encephaloid Structures—Anatomically—Chemically.*

As I have denied the power of any one of the so-called causes of scrofula to produce the disease, it may reasonably be asked whether I am prepared to substitute for them any more probable exciting cause. I believe, that with the exception of the ordinary contagious diseases, and those caused by violence, the greater number of the complaints to which mankind is liable may be excited by a variety of causes, and that frequently several are in action at the same time. When we see the child of persons in easy circumstances suffering from scrofula, whilst the parents show no manifest indication of being the subjects of the disease, or of having suffered from syphilitic taint—when the sufferer does not present the lymphatic temperament, is well fed and clad, and living in large and lofty rooms—we have a difficulty in pointing out a cause; but usually it is not so. Ordinarily, we shall find that such a child has suffered from worms, has bad digestion, increased at the commencement of the second dentition—that there is an indisposition to run about with other children—that its flesh becomes flabby—and that swellings of the glands of the neck are observed. There are two schools not far from my residence, in which I have been enabled to make the following observations:—The number of children maintained in each is about a hundred—the annual admissions about thirty; the dormitories contain about twenty. In the one, the rooms are low and small; in the other, they are large and lofty—in each case infinitely superior to the homes from which they came; in the former of the two schools the cases of scrofula amounted last year to twelve; in the latter, to five. Now when those children were at home, during much of the day the heaven was their canopy—they were running about the streets; they were in the way at home; they were ill fed and miserably clad—for sixteen or eighteen hours they were confined to their narrow homes; for six they were wandering about—but no scrofula was developed. They come to school; they are fed with a sufficiency of wholesome food; their persons are kept clean and well covered; they live in rooms where all possible ventilation is attended to: but mark the difference between the large rooms and the small ones. In these cases, their condition, save and except upon two points, that of breathing the unconfined air of heaven, and using considerable muscular exertion, is greatly ameliorated. What, therefore, prevented the earlier development of the disease? I apprehend these two causes.

I will support this position by further evidence. What caused the lesser frequency of the disease in the children of the Wiltshire peasants than in the child of the Marylebone mechanic or labourer? The more uncontrolled use of legs and lungs—even in spite of worse food. Again, take the children of mechanics in the northern counties of England. What is their life? During much of the day they are occupied in manufactories, not in close rooms, but large halls—and one of two things happens. Either they are leading a sedentary life, unchanged from day to day, or they are exhausted by muscular exertion or *ennui*—in all, the same condition of system is produced; they become flabby and exsanguined, and scrofula is the consequence. Here the air they breathe has not undergone that vitiation upon which M. Baudelocque so much insists, but the result is the same. Take another class of people, in whom the result is still more painful, persons also engaged in manufactories: those who, like the class found in Spitalfields, perform their labour in their own miserable habitations; many members of such families frequently do not go out of doors for days; the children are equally employed. When cold weather comes, to save coal and maintain warmth, every crevice in the window is carefully stopped up, and the door kept shut; here those two causes are in action in all their intensity, and the havoc which this disease makes under these circumstances is very fearful.

At the same time, therefore, that I admit that hereditary predisposition may possibly be entailed—that the child of parents whose health is deteriorated by scrofula or any other disease, may come into the world a miserable and ready recipient of any disease—at the same time that I admit that the *lymphatic temperament* is a probable indication of a constitution less able than others to resist the inroads of disease; that a diseased nurse may furnish milk ill adapted for the purposes of nutrition, capable of disordering the bowels, and perhaps exciting mesenteric disease; that parents who are tainted with syphilis are less likely than persons in health to give birth to healthy children; that food may be so bad in quality, or deficient in quantity, as to produce a general deterioration of the powers of life; that filth and insufficient clothing may materially interfere with cutaneous exhalation; that a vitiated air does necessarily but surely cause a decline of the vital powers; and that the want of muscular exertion brings about a similar condition; yet I have no evidence sufficiently conclusive to produce a conviction on my mind that either of these "causes," acting singly and alone, but uncontrolled as to duration or intensity, is capable of generating scrofula in a person free from the disease when the influence came into action.

*Treatment.*—Now, as to treatment, I would say, if a child is residing in a town, no matter whether in a large house or a small one, a change to the country is very desirable. The change of air, joined to the exercise in the open air which may there be taken every hour in the day, is unquestionably a very powerful means of preventing the development of the disease. With respect to the necessity for change of climate, I have much difficulty in pronouncing. Many circumstances would seem to warrant the opinion, that such changes may exercise a remarkable influence in the development of scrofula. It is unquestionably a fact, that men apparently exempt from all scrofulous disposition or affection, are now and then attacked, when they quit a warm country to inhabit a cold one; and in these cases it is said the disease is more serious and more difficult to cure: the broad fact may be true; but we want to know what has been the change in their habits as well as in the climate. Again, it is not easy to ascertain whether in youth a tendency to the disease was manifested. If a child be brought to you suffering from the disease, and you ask the parents whether they have suffered from a similar affection, you may be sure they will say no, and will vaunt the excellence of their constitution; they may say, "probably the nurse may have been tainted, or that their child has mixed a good deal with some children who had suffered." It is certain that animals transported from warm to cold climates ordinarily suffer from tubercles; but then it is difficult to estimate the influence of climate in producing the disease; their accustomed exercise in searching for food is lost, and they are the denizens of a narrow space, boarded on three sides, so as to allow of a minimum ventilation. On the other hand, it is certain that persons evidently scrofulous are frequently much benefited under the influence of warmer climates; in fact, it seems to be by this circumstance that we are enabled to explain the amelioration which patients undergo during summer, whilst, during other portions of the year, the disease may resist every kind of treatment; and without seeking to depreciate the merits of iodine, every one who has been accustomed to administer the different preparations of this medicine must have observed how comparatively inefficacious they are in the cold months; how decidedly advantageous is their exhibition during summer.

Before we proceed further it is necessary to inquire whether there be any other particular circumstances under the influence of which remedial means present a better prospect of success. As to food, the course I am accustomed to pursue is, to afford my patient what is termed a generous diet, when there is no decided mesenteric affection to contra-indicate it; to give them a moderate quantity of animal food once a day, with well-cooked vegetables, and good bitter table beer, or wine and water. As to cleanliness, this must be carefully attended to, either by means of bathing or sponging; the surface of the body should be daily ablutted and rubbed for some minutes, until thoroughly dry, and the capillary circulation of the surface be stimulated by means of warm towels.

A most important element in the treatment, and one which cannot be too much insisted on, is exercise; but it must not be that kind of gentle exercise which invalid children left to themselves are too apt to take, but such as will largely employ the muscular system; they should be taken out twice or thrice daily in winter, if possible; and in summer they should be very little in the house during the day. It is necessary that games should be provided for them, so as to secure active motion for as long a time as the patient can bear it without fatigue. Indeed, I hold this to be one of the most decided preventives of this disease. I am so strongly impressed with the value of this agent, that I willingly subscribe to an opinion I have somewhere seen maintained, that by the well-directed employment of strong muscular exercise, many cases of this disease, where even tumours are found in the neck, may be cured. I hold it, therefore, to be necessary, that the several means to which I have now alluded should form the ground-work of our treatment of this disease, to which should usually be added the exhibition of certain medicinal substances.

Various medical systems have been greatly eulogized by their respective inventors. Many of them have long been consigned to oblivion and probably some of those still retained, might, without loss, share a similar fate. I have fairly tried four of these systems, and I shall lay before you the results; those which I have employed are the antiphlogistic; in which I include the use of purgatives, emetics, blood-letting, and counter-irritation, the alkaline treatment by the various preparations of iodine, and the mercurial.

Purgatives are unquestionably useful when conjoined with the general means to which I have alluded, and will frequently very manifestly modify, if not cure the disease, and they are especially valuable as an adjunct to the other modes of treatment; they are particularly useful when given during those periodical interruptions which are necessary in the treatment by iodine. How they exercise this beneficial influence is not so easy to explain—whether by exciting the action of the stomach and intestines, by procuring serous evacuations, or by other means; so much, however, is certain, that they are often of great use, and especially as an accessory means of treatment. The impression produced upon my mind is, that those purgatives are most beneficial which procure fluid evacuations, those into which saline substances enter.

My own experience dose not enable me to recommend emetics with so much confidence as seems to have been felt by Bell, Smyth, Bordeu, Kortum, or Dussassoir. Undoubtedly, scrofulous children very commonly present a furred tongue, which is often not cleaned by the use of purgatives; such a case is often much benefited by one or two emetics; but beyond this my belief in their efficacy does not extend. I have never known the frequent use of emetics to be succeeded by any greater amount of amelioration than is usually experienced from the exhibition of two or three.

I have never known more than a passing relief to result from *blood-letting;* and this might naturally be expected, if it be true (and there is every reason to believe it is) that in scrofula the serum largely predominates in the blood. The abstraction of any quantity of blood must necessarily lessen that proportion, and as necessarily increase the evil which it is intended to remedy. The action of purgatives, when they produce watery stools, is the opposite of that. They occasion the exhalation of a considerable quantity of serous fluids upon the mucous surface of the intestines; and by so much lessen the preponderance of the serum in the blood.

In the last and the preceding centuries it was currently believed that we possessed a power of neutralizing the condition upon which the tendency to abnormal deposites depended; and that power was supposed to exist in the *old subcarbonate of potash, or salt of tartar.* Levret believed that it was capable of rendering all deposites fluid, and that in this condition they might be absorbed or evacuated. Although, in the present day, we are satisfied that such virtues are not found in this substance, yet a sort of vague, undefined impression seems to exist, that it is not wholly useless even in scrofula. The Elixir of Peyrilhé, used in France up to the present day, is a mixture of this substance with infusion and tincture of gentian. In the *Collectania Hauniensis* is a case of rickets, which appears to have been successfully treated by this medicine. Internally, I have given this medicine in small and large doses, in almost every form of scrofula, whether affecting the glandular, the mucous, the osseous, or the fibrous tissue; and I am unable to point out any case in which any small amount of relief which may have been obtained during its use, could be fairly referred to this medicine.

In 1784, Crawford proposed as a remedy the *muriate of Baryta;* it was well received, and was very generally used through the greater part of Europe. Suddenly, two very opposite opinions were propagated with regard to it: one, that it was a useless addition to the materia medica; the other, that it was an agent of great energy, and that its exhibition, unless very guardedly, was not without considerable danger. These opinions were no sooner published, than its use was abandoned, without, as it appears to me, any fair trial, in every country of Europe, except Austria. The Austrians were satisfied that in this medicine they possessed a very valuable agent in the cure of scrofula, and my own experience has convinced me that they were right, and that with the exception of iodine, no medicine seems to exert a more decided influence over scrofula than the muriate of baryta. It usually increases the appetite to about as great an extent as we see in children who are taking moderate doses of iodine; it increases all the secretions, and sometimes, like some of the forms of iodine, produces diarrhœa. In twelve cases where it was exhibited in the dose of, at first, one-third, and afterwards half a grain, three times a day, no unpleasant symptom was developed. Eight were materially benefited by its employment. The general health improved sensibly, and the enlargement of the glands was very considerably lessened. In the other four cases, no sensible influence was exerted over the disease. At the same time, however, that I am fully sensible of the valuable character of this medicine, I am bound to admit that its curative effects are less powerful—less certain—than those of iodine, and therefore for some time I have ceased to employ it. Several times I have proposed to use it alternately with iodine, or, when it has been necessary, to intermit the employment of the latter; but I have not yet carried this intention into effect.

Iodine, in its various forms, I have used extensively; and I have had very ample opportunities of estimating the relative merits of the different preparations of this substance. I have administered it in the form of tincture mixed with water, and also associated it with the iodide of potassium. I have exhibited the iodides of iron, lead, sulphur, and arsenic. I have employed it externally, in the form of ointment, lotion, tincture, and bath, and as a broad or wholesale result, I may state shortly, that at present I rarely use internally any other form than the iodide of iron, and that the dose does not exceed, in any case, three grains three times daily. I do not object to the tincture, because, as is alleged, the iodine is thrown down in a pure state when dropped into water, and so applied to the mucous membrane of the fauces and stomach, and is apt to create irritation there; but because I found that diarrhœa was an occasional consequence of its use—that it was inconvenient to trust the persons ordinarily found about patients to administer it—or because, when mixed in considerable quantity, a certain portion is precipitated, and because I found in the ioduret of iron, a more valuable and certain remedy. I am quite ready to admit that many of these inconveniences were lessened by the combination of iodine with the iodide of potassium, suggested by M. Lugol: but the objection I found to attach to this form of administering the medicine, was the bulk of the vehicle, which very frequently disordered the stomach; and when I have lessened it, I have usually seen disorders of the stomach and intestines as a consequence. And in several cases, although the doses have always been moderate, the poisoning effects of this medicine have been developed; and I have no doubt that these effects would have been more frequently seen had we not, from time to time, interrupted the treatment.

Internally administered, I have had no reason to speak strongly in favour of the iodides of mercury, lead, and arsenic. The first and last are unquestionably energetic preparations, but I think them better adapted to certain obstinate diseases of the skin than to scrofulous tumours; and even externally, except in a very dilute form, when they may unquestionably second the internal administration of the medicine, should the quantity of biniodide of mercury not exceed ten grains to the ounce of lard, or the irritation excited upon the part where it is rubbed will be such as to prevent our continuing it. The preparation of lead, in the proportion of a drachm to the ounce of lard, rarely excites similar irritation.

I have a register of two hundred and thirty-two cases in which I have exhibited the iodide of iron. The minimum dose has been a grain twice a day, the maximum three grains three times a day. Of these cases, only three times was it necessary to intermit the use of the medicine for a few days; in one of these it excited ptyalism; it was laid aside for a fortnight, again resumed, and again produced ptyalism. Since that period, and within the last twelve months, the same patient, on her return from Margate, has been taking the medicine with the most decided good effects, and without ptyalism. In the other case, diarrhœa supervened; the medicine was withheld for ten days, was then resumed, continued for several weeks, and without any derangement of the bowels. About once a week an aperient or purgative is given, which decidedly assists the treatment, but no other suspension of the medicine occurs. Where scrofulous ulcerations occur, whether as a consequence of abscess or from other cause, I am accustomed to employ, with the very best effect, a lotion containing three or four grains of this preparation to the ounce of distilled water.

In the employment of oidine or the iodide externally, one fact cannot escape a superficial observer, and that is, the rapid change which follows the application. For a few days this diminution is very striking, but it is not long continued, and after a fortnight or three weeks the tumour appears stationary. Then is the time for resorting to a new form, which must be employed for a similar period, and must then give place to a third. But although these external applications will occasion a marked diminution of such tumours, they hardly ever completely disperse them; and when applied alone, without a concurrent internal administration of some preparation of the medicine, their effects are much less decided. When such tumours are extremely indolent, the ointment may be rubbed upon the part without fear of injury; but if they be the seat of irritation, it is very likely to be increased by friction. In consequence of this circumstance, I usually recommend it to be applied, to the part, spread on lint. It is thus kept in contact with the surface for a much longer time, the irritation consequent upon rubbing is avoided, and the good effects of the medicine are more decidedly marked than by any other mode of application.

Many authors speak of great or partial emaciation consequent upon the use of iodine. Jahn describes cases in which the emaciation was general. Coindet has referred to a diminution of the mammæ. Hufeland also gives three examples of it. Others have referred to the testicle as suffering in a similar way. And these isolated cases, which may or may not have succeeded to the use of iodine, are erected into a general law. Now, in my own experience, so far from emaciation of the whole or a part of the body being essential to the therapeutical action of this medicine, when prudently administered, one of the earliest symptoms observed is a remarkable increase of appetite, and a corresponding increase in the bulk of the body. I have watched its effects with great care, and I have not known a single case in which either the whole or even a part of the natural structures of the body have undergone any such change.

My experience of iodine in the form of baths is inconsiderable: such as it is, it leaves no desire on my mind to extend it. In two cases a considerable and troublesome eruption on the skin was produced; in three cases vertigo, with a suffused countenance, was occasioned, which was not dissipated for many hours, and no sensible good effect was produced on the tumours. These circumstances, added to the costly nature of the remedy, have deterred me from prosecuting further this mode of treatment. I know that this opinion is in opposition to that of Lugol, who is satisfied that the cure of these diseases is much accelerated by the conjoint use of baths and internal remedies; but any one who reads the cases given by Lugol cannot fail to recognise the same effects which I have described, though with less intensity. However, Baudelocque has come to a conclusion not very different from my own. Still he points out a remarkable effect which he has observed upon suppurating surfaces: he has always seen the suppuration much diminished, and the surface contracted: so that for some days much less linen was required for dressing the patients; but this effect does not seem to have been permanent.

Relying upon the encomiums of Hufeland, Chermeil, and others, I resorted to the use of the *black sulphuret of mercury* in the treatment of this disease; but, whether associated with hemlock, magnesia, or ipecacuanha, I found no sufficient reason to induce me to employ it generally. I do not deny that the disease is often gradually but slowly ameliorated during the administration of this medicine; and I have never known any unpleasant effects, such as salivation, to result from its use; on the contrary, the tongue and the evacuations have improved under it, but with much less certainty and a much greater loss of time than under the influence of iodine. I prefer it to the common mercurial remedy employed in such cases—calomel and rhubarb—because, with the exception of the amount of good produced by evacuating the bowels, I have never seen any decided antiscrofulous virtue manifested by it.

Though, under the influence of those remedies which we have just been considering, a patient's general health may be very decidedly improved—though glandular tumours may lessen—and even where suppuration has taken place, and the integuments over it have become thinned, they may be dissipated, yet where scrofulous matter has been deposited in its cheese-like form, neither iodine nor any other remedy which we know has power to procure its absorption; where it is deposited there it must remain; a point around which irritation is easily kept up, and about which, sooner or later, suppuration will take place; the abscess will either break, or art will interpose to facilitate this result by puncture, and it may thus be eliminated from the system. True it is, however, that the disposition to deposite this matter may be neutralized, and that all the more fluid portions of matter so deposited may be absorbed, and that, after death, a mass of cretaceous matter will be found to occupy its place. But in a large number of cases, spite of the most prudent treatment, the local disease will end in abscess; for instance, out of eighty-nine cases, thirty-three presented this termination. When this is inevitable, it is unquestionable that we ought to anticipate by puncture, or other means, that gradual thinning of the tissues to which nature resorts in accomplishing the object; but it is not an easy matter to determine whether or not a scrofulous abscess will advance or retire: we may see the integuments so thinned that only the cuticle would seem to prevent its emptying itself, and yet it will retire—the whole of its contents will be absorbed. It must, however, be borne in mind that such abscesses are usually found to occupy the cellular tissue, and sometimes a lymphatic vessel, where no gland exists; in those cases where the abscess surrounds a gland where the product deposited in the substance of the gland is a constant source of irritation, the onward progress of the disease is more probable. It would, of course, be desirable that not only the thin sero-purulent matter which is usually contained in such abscesses, but also the scrofulous product, should be evacuated before the thinning has proceeded far, and the violet colour of the integuments is displayed, but this is a desideratum not easily accomplished. If the product have not undergone softening, often no evacuation of the matter will take place; if it have, a slight oozing, bringing away, from day to day, small portions of this matter, will be the cause of evacuation, and often many months will elapse before the gland and its contents shall have been evacuated; and at the end of that time an unsightly cicatrix will be the consequence. This result is accomplished in the following way:—one or two small openings in the thin violet-coloured integuments are the channels through which the matter is discharged. A more or less extended cavity exists under, produced by the breaking down of the gland and its surrounding cellular tissue. When the whole of this structure is broken down and evacuated, this surface presents granulations which have a tendency to skin over without adhering at all, or on other occasions only partially, to the superjacent thinned integuments. The consequence of this is an irregular puckered surface; and when, as is often the case, the subjacent tissue becomes adherent to the deeper-seated organs, the deformity is increased by a pitting. To prevent this aggravation, two modes may be resorted to. When the time for procuring the evacuation of such a tumour has arrived—when the integuments have become much thinned—the best mode of opening it is by applying the Vienna caustic paste to the part, taking care that the paste shall include the whole of the thinned structure. A fair and sufficient opening will be thus made; the evacuation will be more speedy, the remaining tissues will be healthy, and the cicatrix will be comparatively trifling. If, however, this have been neglected, or another course pursued—if the discharge be going on from one or more small points—if the integuments over the parts be very thin—then with scissors excise the whole of the violet integuments, and you may hope to lessen the deformity which would otherwise succeed to the disease. But much valuable time would probably be lost in the endeavour to heal the sinuses connected with the cavity; the various forms of iodine, in a more or less concentrated state, would have been applied to them, and the patient subjected to much suffering. And here I may state that after ample experience of such applications to these sinuses, I am decidedly of opinion that they occasion more pain and are much less efficacious than the nitrate of silver.

### CANCER.

*Definition.—Nature.*—When the nature of a disease is unknown—when it presents much variety in appearance and symptoms, changing with the period of the disease, and the organ affected—it is perhaps impossible to give a precise definition, which shall comprehend the many changes of its existence, and embrace all its varieties. Such is cancer. In the present state of science cancer appears in a large majority of cases to consist in a diathesis, in virtue of which certain new structures are developed, which may be indefinitely extended, which tend to ulceration, and which lead to the destruction of life, either by interfering with some important function, or by producing general exhaustion. In other words, we may call the cancerous diathesis a constitutional disease, manifested by the deposition, at one or more points of the economy, of an abnormal product.

Whether the term cancer was introduced into medical science from a fancied resemblance between the dilated veins which often stretch along under the integument of a cancerous tumour and the claws of a crab, or because the patient usually experiences a sensation which has been compared to that which would be produced by tearing or gnawing by the same animal, we shall not stop to inquire. It is sufficient for us that it is a well-understood term applied by the Greeks to certain tumours of the breast, and which in after times has been extended to similar tumours in other parts of the body. In preserving up to the present moment an expression so eminently vicious, the moderns have attached to it much more precise ideas than the ancients could, ignorant as they were of pathological anatomy. Still, science is not as yet sufficiently advanced to enable us to resolve many questions relative to cancer, which in many respects, unquestionably, is one of those diseases, the history of which imperiously requires new observation and research. The structure of cancinomatous products is at the present moment a favourite object of investigation, and we may therefore hope that our knowledge of them will ere long be considerably extended.

Persons of both sexes are subject to it, but women most frequently; in the latter, from forty to fifty is the period of life during which it is most commonly observed; still the exceptions to this rule are many. In many cases we may observe what is known as a bilious temperament, a morose or melancholy character, a highly developed sensibility and irritability; whether these circumstances are true predispositions to cancer, or whether they should be considered only as proper to favour the action of an internal cause capable of producing the disease, is doubtful. We cannot tell what is the influence of age, sex, and constitution upon the disease; we know only that in woman the return of the menstrual period exposes the cancerous tumour to a sort of erethism or periodical orgasm, under the influence of which its growth is greatly accelerated; and that when the function has completely ceased, the suppression of the hæmorrhage often impresses upon the disease a more rapid course.

Cancer is one of the most painful, the most incurable, and most frequent, of the diseases by which humanity is afflicted. Its course is always onwards—it never retrogrades; a cancerous tumour being, so far as I know, unsusceptible of resolution. It converts adjoining parts into a tissue like its own, and when left to itself, or even when art interposes, is almost always fatal.

*Manifestation.*—Most commonly, without known cause, sometimes as a consequence of violence, or slight irritation, a general or partial tumefaction is manifested in some organ, or as an independent tumour, which, from its size, its form, and its relations, can scarcely be confounded with any existing organ. Sometimes, from the first, the tumour is painful, sometimes so sensible that the slightest touch is insupportable, sometimes it is completely indolent, and only remarkable for its volume. In the latter case it may become very large before much pains are felt. Whatever the extent of their development, these tumours are usually hard, unequal, lobulated, and heavy; sometimes they are soft, elastic, and apparently fluctuating. So marked may they be, that the tumour has been mistaken for a cyst containing fluid; in this way a fungus hæmatodes of the breast and a pulpy testicle have been punctured. Left to itself, it increases, approaches the skin, adheres to it, produces many changes of colour, thins it, and ulceration follows. The peculiar appearance of the ulcer, when it occurs, and the lancinating character of the pains which accompany it, are regarded as sufficiently marked to enable us at all times to distinguish the disease; yet they are fallacious signs. An enormous cancer tumour has become the seat of gangrene, and the ulcer has healed, and an ulcer resting on a cancerous base may cicatrize. The lancinating pain is felt in other diseases than cancer, and certain internal cancerous masses may never ulcerate. It is therefore necessary to inquire at once into the anatomical characters which are peculiar to these diseases, as the only safe means of diagnosis.

*Anatomical Characters—Varieties.*—The structures included under the term cancer are various, but in their course and their results there is considerable uniformity. The common *vascular sarcoma* of Abernethy, presents an appearance not unlike that of a mass of fibrin which has coagulated in the vessels, lost a portion of its colouring matter, and become organized. A similar product is sometimes cellular, and in these cells a serous fluid is contained; this is the *cystic sarcoma* of the same author. In other cases the diseased tissues are granular, bearing a resemblance to the pancreas; this is the *pancreatic sarcoma* of the same author. When the morbid product is presented under the form of a grayish or whitish substance, exhibiting no trace of vessel or blood, frequently divided into lobules, by fibrous intersections, which grate under the scalpel, it is termed *scirrhus*. When it presents an appearance like the cancerous tubercle of the liver, it is termed *tuberculated sarcoma*; when it resembles the appearance of the mammary gland, it is termed *mammary sarcoma*. When the substance resembles the substance of the brain, it is termed *encephaloid* or *medullary tissue*. And when the latter tissue softens, and at points acquires great vascularity, the vessels giving way, and blood being sometimes extravasated, and presenting a bloody fungous mass, it is termed fungus hæmatodes. Carswell divides carcinoma into two species; the distinction between which he founds upon the greater or less disposition to become organized; and supposing that to be well made out, it is a most important distinction, because their tendency to degenerate bears a very exact relation to their vascularity: those are *cephaloma* and *scirrhoma*. In the former he includes schirrhus, the pancreatic sarcoma of Abernethy, the tissu lardacé of French authors, the matière colloïde of Laennec, the gelatiniforme or aréolaire of Cruveilhier. In the latter he includes the common vascular or organized sarcoma, the mammary, and the medullary sarcoma of Abernethy. He admits, however, that it is often impossible to draw a distinct line of separation between them, and this we cannot be surprised at, when it is shown, "that examples are not rare of scirrhus, medullary sarcoma, and fungus hæmatodes, originating in the same morbid state, and passing successively from one into another, in the order in which they are named." If this be so, those distinctions may appear to some persons of little importance. Yet if the curability of the disease be dependent to any extent on the time at which the remedy is applied, such distinctions must be desirable. If, as some persons believe, the reproductive tendency varies with the particular structures, it is imperative upon us to endeavour to make out such distinctions as clearly as possible.

Müller makes a different arrangement of these structures. He distinguishes them into carcinoma simplex or scirrhus; carcinoma reticulare, which seems to be a variety of the former, the reticular arrangement being more decided, it is, says he, as frequently seen, if not more so, in the female breast than carcinoma simplex; carcinoma alveolare, which is the cystic or cystiform of some authors, the aréolaire or gelatiniforme of Cruveilhier; carcinoma melanodes or melanosis; carcinoma medullare, which is the medullary sarcoma of Abernethy, encephaloid tissues of Laennec, sprungoid inflammation of Burns, fungus hæmatodes of Hey, Wardrop, C. Bell, soft or spongy cancer of Roux, milt-like tumour of Munro, medullary fungus of Maunoir. The remaining variety he describes is carcinoma fasciculatum, which is very rare. The greater number of modern pathologists regard these various structures as non-analogous or heterologous formations; and if we regard heterologous formations as depending upon the presence of a structure which does not enter into the healthy composition of the body, we can scarcely object to the term as applied to these products. However, so great an authority as Müller regards them as analogous formations; he says the finest parts of these structures do not differ from parts of other innocent tissues, or primitive formations of embryo life. This may be very true, but do we find the same elements in combination in natural structures? I apprehend not; and therefore they are tissues which have no "analogue" in the healthy structures of the living body.

We shall limit our considerations to three classes of cancerous structures, scirrhoid, medullary and melanotic formations, of which the two former constitute, in a large proportion of cases, incurable diseases. We shall first describe scirrhoid and encephaloid diseases; space will not admit of our entering into the particular history of each of the morbid structures comprehended in the term cancer, and therefore we must be underderstood to apply our remarks more to a class than to species.

*Scirrhoid structures.*—Although the difficulties of definition be great, we may say that scirrhus is commonly presented in the form of a hard, and usually unique tumour, little sensible upon pressure; from time to time the seat of darting, lancinating pains, occurring without obvious cause, generally making very slow progress: rarely occurring in young persons. Sometimes appearing stationary for twenty or thirty years; at others, where it makes progress, it is still so slow that many years may pass without very sensible enlargement. Scirrhus has a marked predilection for what may be termed white tissues. Either it supervenes spontaneously, or succeeds to a congestion, from external or internal cause. It is most commonly presented at that period of life when the power of reproduction ceases. It is often manifested after long mental discomfort; its development seems to be favoured by inaction. Once formed it never retrogrades, and the affected part never resumes its former condition. In this respect it differs from simple induration resulting from chronic inflammation. It may be stationary for many years, determining no discomfort, but suddenly it manifests activity, and a cancerous mammary gland, which has been indolent for ten or fifteen years, becomes the seat of intense pain and rapid disorganization.

*Anatomically.*—Scirrhus, when cut through, may present considerable variety in appearance; it may be very resistent, grating under the scalpel, of a bluish white or gray colour, and semi-pellucid. Its general appearance bears considerable similarity to that presented upon section in a radish. If carefully examined, we find it to be composed of two substances—one opaque, fibrous, forming intersections so as to constitute septa or areola, which contain another substance, which is more or less diaphanus—lard-like, horn-like, or semi-fluid: this may often be squeezed out. Sometimes those white septa extend beyond the tumour, and Sir C. Bell insisted much upon the influence they had in the reproduction of cancer. Unless all these radii were removed, relapse was, he believed, inevitable; this was also the opinion of Abernethy. Sir C. Bell believed that these septa were developed first, and that the softer matter was afterwards deposited in the spaces circumscribed by these septa. In scirrhus, before softening, the vascular system is at its minimum of development; Scarpa attempted to inject this structure, but he found that, though the arterial tissues immediately around were well injected, no injection penetrated into the tumour. Rouzet, after the most minute examination, could not discover in scirrhous structures any blood-vessels. Scirrhous rarely acquire the bulk of encephaloid or medullary tumours, nor do they possess their elasticity and decided lobular character. In some cases the existence of scirrhus in an organ produces atrophy; this is occasionally seen in the breast, the testicle, the spleen, and the kidneys; that is, it seems to excite the absorption of the cellular tissue of the organ. Scarpa believed that scirrhus is never primarily developed elsewhere than in the external conglomerate glands, the tegumentary, and in certain portions of the mucous membrane;—the glands in which it is most frequently found are the mammary, the parotid, the testicle, the submaxillary and the lachrymal; the mucous membrane of the œsophagus, the stomach, the rectum, the vagina, the neck of the uterus, the larynx. This is the opinion of a man of great experience, but cases may be adduced in opposition to it, for this product has been seen on the pleura and other analogous situations. However, the cases are not, I think, sufficiently numerous to confirm that law of the development of scirrhus, which Scarpa laid down. When it extends, it gradually affects the adjoining tissues, slowly approaches the skin, adheres to it, but before ulceration takes place certain changes occur, it is generally softened, and assumes at some points an appearance somewhat like that of medullary fungus.—When softening has proceeded to some extent, the physical characters of the structure are much changed; it becomes softened at many points, and assumes the appearance of a semi-transparent jelly, sometimes of a grayish colour, sometimes slightly tinged with blood. So that in many cases one or more cavities exist before the skin gives way. The skin soon reddens and cracks, which is the ordinary beginning of ulceration in scirrhus; the ulceration extends, the surface is irregular, often dry, grayish, red or brown; at other times it is covered by fungous matter. If we cut through it, we see that the floor presents a fleshy appearance, very friable, easily broken down with the nail. Hæmorrhages are not frequent in ulcers succeeding to scirrhus, and when they occur are usually not abundant, unless an artery have given way.

*Chemically.*—The only very precise chemical analysis of scirrhus is that of Hechtfils, from which it resulted that scirrhus of the female breast yielded gelatine, fibrin, oleïne, some traces of albumen, and water, in about the following proportions:—

| | |
|---|---|
| Albumen, - - | 2 |
| Gelatine, - - - | 20 |
| Fibrin, - - - | 20 |
| Fatty matter, (fluid,) - | 10 |
| Water, and loss, - | 20 |

And from a similar analysis of scirrhus of the uterus, it resulted that it contained no albumen, but afforded gelatine, fibrin, and fatty matter, soluble in alcohol, in the following proportions:—

| | |
|---|---|
| Water, - - | 35 |
| Fatty matter, - - | 10 |
| Fibrin, - - | 10 |
| Gelatine, - - | 15 |

Analyses have also been given by Berzelius and Müller.

*Medullary structures.*—Medullary or encephaloid matter is, in colour and consistency, not unlike that of the brain of young children. Before softening has proceeded far, a section of the structure presents an almost homogeneous, pulpy matter; the colour is pale or slightly rosy, but it is never uniform; here and there redder points are discovered, and there the softening is more decided, as well as the vascularity; occasionally much darker points are observed, produced by the rupture of the very delicate blood-vessels of the structure, the blood being mixed up with the brain-like matter, and giving to it a reddish, brownish, or darkish character. In cases of icterus, the medullary colour has been seen of a yellowish character. Before any considerable change is produced, the colour is usually what I have described it. Like the substance of the brain it is reduced, by contact of air, to the condition of a semi-fluid pulp; like it, is miscible with cold water; like it is hardened by alcohol and acids. It is, however, the fluid portion which is thus changed: this matter, between the first and second period, is milky; but then it cannot be obtained by compression, it is necessary to scrape the surface with a scalpel; at a later period it can be expressed by compression. This is the medullary or encephaloid matter—this it is which is found in veins, and may be squeezed out of them.—These tumours may acquire great size. Abernethy describes a case, where in each groin was a tumour as large as the head of an adult—the medullary tumours acquire the greatest bulk in the limbs. A case is stated by Olivier, where such a tumour on a woman's thigh attained the bulk of a man's body. In Gooch's case the size of the tumour may be estimated by the fact, that a line drawn directly from the elbow to the wrist, measured four feet (Cases, &c., p. 39.

The opinion supported by many persons, that medullary structure is only an advanced stage in the development of scirrhoid structures is, I think, much too exclusive. We have seen that at an advanced period of their existence the differential characters are easily pointed out; in an earlier period these distinctions are less decided. The fineness of medullery tissue at its first stage may be equal to or greater than firm lard, and its vascularity is not much developed; at this time, though similar, they are not identical. The softening of the medullary structure is not like that of scirrhus. Trousseau and Leblanc, say that the granules of medullary matter are larger and their consistence less than that of scirrhus; that, under the knife, medullary does not grate like scirrhous structure. It is true that the scirrhoid structures do in many cases undergo changes, which more or less completely identify them with medullary structures, but it is quite as true, that generally medullary structures are medullary ab initio.

*Progress.*—Encephaloid structures grow with great rapidity. Cases are mentioned by Andral where, in internal organs, fourteen to thirty days only have elapsed between the occurrence of the first symptoms and a fatal termination of this disease. But these are unsatisfactory and inconclusive illustrations, because it is impossible to ascertain to what extent this disease was developed upon the occurrence of the earliest symptom. Still many long years may elapse; in the case described by Gooch, fifty years. But there, again, we cannot say at what period the structure assumed an encephaloid character. At or near the surface, its progress is more rapid than when deeper seated; it is not usually a uniformly round tumour; as it increases it is irregular, and projects at one or many points, and in these points its consistency is diminished; there is an elastic softness which simulates fluctuation, the cutaneous veins are varicosed, the skin becomes erysipelatous, ulcerates, and red and bleeding fungous matter is developed; it then assumes the character of fungus hæmatodes. At this time, if we make a section of the structure, it presents a rosy colour, much deeper at some points than others; when ulceration takes place, hæmorrhage is frequent, and portions of the mass come away in a half decomposed state. A thin, fetid, sanious fluid escapes, and the progress of the disease is rapid: many years may have passed before ulceration, sometimes a few weeks or even a few days are then all that remain of life; large masses sometimes come away, and large excavations succeed to them, but it is very rare that any attempt to cicatrize is shown. Abertheny thought that as these structures increased in bulk, they rather pushed aside than converted the adjoining tissue into their own character; and this he considered the great distinction between medullary sarcoma and scirrhus. This opinion is too exclusive, because medullary structure may be propagated by continuity, may convert bone, but most constantly it causes the absorption of these organs; venous parietes seem to give way easily, arterial tumours often remain long unaffected in the midst of masses of encephaloid matter. Still arteries will now and then give way, and occasion fatal hæmorrhage, and veins will sometimes resist for a long time. Velpeau describes a case of a medullary mass in the axilla which ulcerated, and the patient lost nine pounds of blood in twelve days. A similar case is mentioned by Abernethy. Fibrous tissues, such as aponeuroses and tendons, seem to resist effectually, and sometimes constitute an effectual barrier to the extension of the disease. Besides extension by contiguity, the disease may invade distant tissues—a pulpy testicle will affect the lumbar glands with a disease like itself; now whether this be by the transport of medullary matter from one organ to the other through the medium of the veins or lymphatics, or whether a simple irritation be communicated, and, the system being deteriorated by the disease, carcinomatous matter is deposited there, may admit of question. So much seems evident; the disease of the testicle may exist for a long time without affecting the lumbar glands, and merely does so before the system gives indication of being impregnated with the disease. It is true, the general infection of the system may be a consequence of absorption of the morbid matter. In scirrhus we do not often find masses of the disease in different organs.

[*Medical Gazette.*

---

*Sea Scurvy.*—The following is a well-marked instance of a disease which has been rarely seen since the health of seamen has been promoted by attention to the cleanliness, dryness, and ventilation of the ships, by procuring a regular supply of lemon-juice and vegetable food, lessening the length of watches, the better preservation of water, and the diminished consumption of ardent spirits. By these means scurvy, which not unfrequently destoyed whole ships' companies, has been so far subdued, that there are many medical men who have never seen a single case, and it is only now, when the present objectionable mode of paying the captain a certain stated sum for working the ship, tempts him to enrich himself by injuring his crew, that we get an example in the hospitals. It is to be hoped that the circumstances connected with this case will have some effect in suppressing a practice so likely to prove injurious.

Archibald M'Donald, aged 28, admitted Nov. 20, 1837, under the care of Dr. Burton.—States that he started from Greenock in October, 1838, in the ship William Nicholl, of Glasgow, Captain John Potter. The vessel is a large one, and "works heavy." There were twenty-one men and two boys, a small crew for the size of the vessel, under any circumstances. The passage out, however, was fortunate, and the health of the crew good, though lemon-juice was not served out so frequently as is common in the service. They laid about two months at Calcutta, and lost four men from some paludal fever, and as no fresh hands were taken in, there were only nineteen, with the boys, to work the homeward passage. There was a good deal of bad weather, and the watches were much lengthened. The captain had bought a stock of buffalo beef, much of which was putrid when served out, and he neglected to supply the crew with vegetables or acids. The consequence was, that a few days before they reached the Cape of Good Hope, the men were suffering from extreme debility and lassitude, stiffness and feebleness of the knees, great fatigue and panting after exertion. The legs then began to swell, and great pain came on in the ankles and back, also pains and a sense of great constriction in the chest. The skin of the legs became in some places dry and rough, the cuticle cracking; in others it was smooth and shining, with blue, red, or black, livid subcutaneous spots or patches. They put in at the Isle of France, where two men insisted on being discharged. Two others were taken in their place, but neither vegetables nor fresh meat were shipped as usual. The disease accordingly went on through the whole crew, hæmorrhages from the mouth and arms became common, and the teeth of the sailmaker, an old man, dropped out. Two men died immediately after the ship reached London, and some judicial inquiry was made into the circumstances, with what effect we are not aware. This man, on admission, presented the dark swollen chapped state of the legs, spongy gums, fœtid breath, and general debility, observed and described by Cook and others. He was ordered an ounce of lemon-juice in decoction of bark three times a day, and diet consisting of fresh meat and vegetables. He has been gradually improving, and is now about to be discharged.

*Lancet.*

---

*New Moxas.*—M. Graefe, of Berlin employs the following simple method of preparing moxas. A wafer is steeped in three parts of essence of turpentine with one part of ether, and then dried with a bit of lint. If a larger one be required, a round piece may be cut out of communion bread (pain à chanter.)

*Journal de Chimie, Dec.*, 1839.

